# Discussing Conflicting Explanatory Approaches in Flexibility Training Under Consideration of Physiology: A Narrative Review

**DOI:** 10.1007/s40279-024-02043-y

**Published:** 2024-05-31

**Authors:** Konstantin Warneke, David G. Behm, Shahab Alizadeh, Martin Hillebrecht, Andreas Konrad, Klaus Wirth

**Affiliations:** 1 Institute of Human Movement Science, Sport and Health, University of Graz, Graz, Austria; 2 Department of Movement Sciences, Institute of Sport Science, University of Klagenfurt, Universitatsstraße 65, 9020 Klagenfurt Am Wörthersee, Austria; 3 School of Human Kinetics and Recreation, Memorial University of Newfoundland, St. John’s, NL Canada; 4 Human Performance Lab, Department of Kinesiology, University of Calgary, Calgary, AB Canada; 5 University Sports Center, Carl Von Ossietzky University Oldenburg, Oldenburg, Germany; 6 University of Applied Sciences Wiener Neustadt, Vienna, Austria

## Abstract

The mechanisms underlying range of motion enhancements via flexibility training discussed in the literature show high heterogeneity in research methodology and study findings. In addition, scientific conclusions are mostly based on functional observations while studies considering the underlying physiology are less common. However, understanding the underlying mechanisms that contribute to an improved range of motion through stretching is crucial for conducting comparable studies with sound designs, optimising training routines and accurately interpreting resulting outcomes. While there seems to be no evidence to attribute acute range of motion increases as well as changes in muscle and tendon stiffness and pain perception specifically to stretching or foam rolling, the role of general warm-up effects is discussed in this paper. Additionally, the role of mechanical tension applied to greater muscle lengths for range of motion improvement will be discussed. Thus, it is suggested that physical training stressors can be seen as external stimuli that control gene expression via the targeted stimulation of transcription factors, leading to structural adaptations due to enhanced protein synthesis. Hence, the possible role of serial sarcomerogenesis in altering pain perception, reducing muscle stiffness and passive torque, or changes in the optimal joint angle for force development is considered as well as alternative interventions with a potential impact on anabolic pathways. As there are limited possibilities to directly measure serial sarcomere number, longitudinal muscle hypertrophy remains without direct evidence. The available literature does not demonstrate the necessity of only using specific flexibility training routines such as stretching to enhance acute or chronic range of motion.

## Key Points

**Table:** 

Stretching and foam rolling are the most common training interventions when aiming to acutely or chronically increase range of motion. However, it seems questionable to attribute the flexibility increases exclusively to these training routines.
The limitations in study methodology are discussed, including different training protocols that result in improved range of motion. This leads to the misinterpretation of the findings based on speculative physiological mechanisms.
Acute and chronic stretching are of limited efficacy. Similar range of motion enhancements can be achieved via other training interventions sufficient to enhance body temperature acutely, while chronically, applying mechanical tension to extended muscle lengths seems one important factor, achievable through concurrent alternatives (e.g. resistance training).

## Introduction

In sports practice, improving physical fitness requires a well-planned and structured training process. Clear definitions and understanding the underlying mechanisms increasing range of motion (ROM) via different methods is a vital prerequisite for developing training regimes. In flexibility research, many definitions regarding flexibility have been proposed [1, 2] which range from “total achievable excursion (within limits of pain) of a body part through its potential range of motion (ROM)” to “ability to move a joint through a normal ROM without undue stress to the musculotendinous unit” [3, 4].

In recent decades, numerous stretching modalities [2, 4–10], in addition to foam rolling interventions [11–17] as well as different massage techniques [18] with their respective acute and chronic effects on flexibility were investigated using different study methodologies and outcomes. Because most of the interventions showed increased flexibility [4, 6, 17, 19], questions arise about possible shared underlying mechanisms between these interventions. Additionally, regarding chronic flexibility increases, there is a vast amount of evidence pointing out the listed stretching modalities as sufficient to increase ROM. Most studies explained flexibility increases via changes in muscle–tendon stiffness and changes in the pain threshold [20] as the most frequently reported explanatory approaches [3, 4, 21–25]. However, because acute and chronic flexibility increases can also be induced via other training routines, this narrative review discusses the similarities and discrepancies in underlying mechanisms between different methods leading to enhanced acute and chronic flexibility and provides explanatory approaches based on a biological and systematic level.

## Overview About the Most Popular Training Protocols and Results to Enhance Flexibility

Stretching can be considered as the most popular modality to increase ROM. Commonly, the literature describes static stretching as holding a muscle in the end ROM at the point of discomfort, while dynamic stretching is described as performing controlled movements in the end ROM or ballistic stretching using bouncing movements [2]. Proprioceptive neuromuscular facilitation (PNF) could be divided into three subcategories, using cycles of static stretching and (sub)maximal contraction of the stretched muscle contract-relax PNF, the antagonist contraction method that uses antagonist contraction with static stretching and the contract relax antagonist contraction method, which combines both methods [2].

### Acute Flexibility Effects

Regarding acute flexibility improvements, the literature provides no clear evidence which stretching technique should be prioritised within a single training session. There is evidence supporting the effectiveness of all stretching types to increase ROM [3, 4, 8, 9, 15, 26–29]. Accordingly, recent systematic reviews (with meta-analyses) have similarly reported no difference between one bout of static stretching and dynamic stretching [30], as well as no significant acute ROM differences between the reviewed stretching techniques, independent of stretch intensity, sex or training level [31]. The literature has attributed acute stretch-induced flexibility increases to enhanced pain (stretch) tolerance [13, 31, 32] possibly due to adaptations of nociceptive nerve endings, as well as decreased stiffness in the musculotendinous unit [33], muscle [19, 34–37] and tendon [38, 39] properties [40, 41], as well as thixotropic effects [13]. Based on these adaptations, stretching was considered an integral component of warm-up routines over decades [42, 43]. However, most current systematic reviews addressing the acute effects of prolonged static stretching (i.e. > 60 s per muscle group) on strength performance did not recommend its inclusion in warm-up routines because of its harmful effects on subsequent performance [44, 45]. Even though the negative influence on performance could be compensated/weakened by adding more active and dynamic warm-up activities to stretching [46], the listed limitations called for alternative routines that acutely improved flexibility without impairing subsequent performance.

Therefore, the literature proposed foam rolling as a sufficient alternative to prolonged static stretching to acutely enhance flexibility. Foam rolling involves small undulations over a muscle with a dense foam wrap around a solid plastic cylinder, rolling from the proximal to distal portion of the muscle or vice versa [2, 47]. The participant usually places the foam roll on the ground or floor, with recommendations to perform 2–4 s repetition duration (time for a single roll in one direction over the length of a body part) with a total rolling duration of 30–120 s per set [48] in order to apply mechanical pressure to the tissue. Foam rolling is proposed to be helpful regarding several aspects, such as increasing flexibility [17], decreasing pain, minimising symptoms of delayed-onset muscle soreness and the release of myofascial adhesions [17, 47]. Therefore, to acutely increase ROM in warm-up routines, the use of foam rollers is frequently recommended [17, 49, 50] with expected ROM increasing approximately 4.0% [11], similar to static stretching [13, 17]. Suggested underlying mechanisms seem similar as well with reported foam rolling effects on muscle stiffness [51–53], or reduced pain sensitivity [54, 55]. Further explanatory approaches suggesting myofascial release would cause, for example, stiffness decreases, and blood flow changes seem not appropriately investigated [47]. While stretching total durations of up to 480 s were described of practical relevance [56, 57], most studies used stretch durations per bout with up to 120 s [45]. For foam rolling, the literature recommends the usage of up to 4 × 60 s [48, 58]. Even though Konrad et al. [13] showed no difference in acute ROM effects with foam rolling, studies that directly compared the dose–response relationship for foam rolling and stretching were not found.

The literature provides further alternatives to static stretching. Interestingly, in accordance with Alizadeh et al. [59], full ROM resistance training can be considered a type of loaded dynamic stretching. However, only a few studies explored the acute effects of resistance training on ROM. For example, Warneke et al. [60] showed no significant difference between acute ROM increases in the calf muscles comparing high-volume stretching (1 h) with an exhausting calf raise routine consisting of 5 × 12 repetitions performed over the full ROM. Most recently, Murakami et al. [61] showed 3 × 10 repetitions of full ROM standing calf raise exercises to be effective in increasing ROM and passive peak torque, while stiffness remained unaffected. Additionally, Oliveira et al. [62] found no acute stiffness reductions in the pectoralis muscle after 3 × 8–13 repetitions of bench press. In contrast, Krzysztofik et al. [63] showed significant stiffness reductions in the Achilles tendon and the quadriceps as an acute response to one to two bouts of barbell squats. Kawama et al. [64, 65] reported significant stiffness reductions in the hamstrings when training was performed over the full ROM. Thus, resistance training also seems to acutely improve flexibility, however, without clear evidence regarding their underlying mechanisms. These are only a few examples, other studies demonstrated flexibility enhancements after using alternative routines such as “neurodynamic techniques” [66–68], flossing [69] and massage with percussive massage devices [70].

### Acute ROM Improvements Due to Enhanced Muscle Temperature?

With the current literature of acute stretching effects, it can be noted that most of the listed activities applied training stimuli on the muscle with similar responses, including reduced pain sensitivity [12, 13, 31], and reduced muscle, tendon or muscle tendon unit stiffness [33]. These adaptations could be induced by improvements in viscoelastic properties and thixotropic effects [13, 47]. However, these phenomena and outcomes are not exclusively attributable to stretching or foam rolling or other specialised interventions.

Most stretching or foam rolling studies included a passive control group. However, these comparisons are only valid to show that both routines can be used to enhance flexibility compared with doing nothing. However, the enhanced flexibility may not be just attributed to the actions of stretching or foam rolling as there may be other alternative explanations. As the listed training interventions improved flexibility compared with an inactive control, these findings merely support the notion that overall activity appears to be sufficient in comparison to a state of rest. However, as general muscle dynamic activity is known to enhance muscle and core temperature (general warm-up effects), which are sufficient to economise muscle contractions by decreasing joint friction, increasing synovial fluid with accompanied decreases in muscle viscosity [71, 72] and increased blood flow [73]. Interestingly, Reid [74] described a warm-up-related improvement in the myotatic reflex, leading to a later occurrence of stretching pain. Consequently, it could be hypothesised that stretching/foam rolling/resistance training effects might originate from general warm-up effects occurring from muscle contraction-induced exothermal ATP splitting while performing foam rolling/dynamic stretching. Even though static stretching electromyography activity was smaller compared with alternative methods [75], Oliveira et al. [76] confirmed temperature increases after performing 180 s of static stretching.

**Fig. 1:**
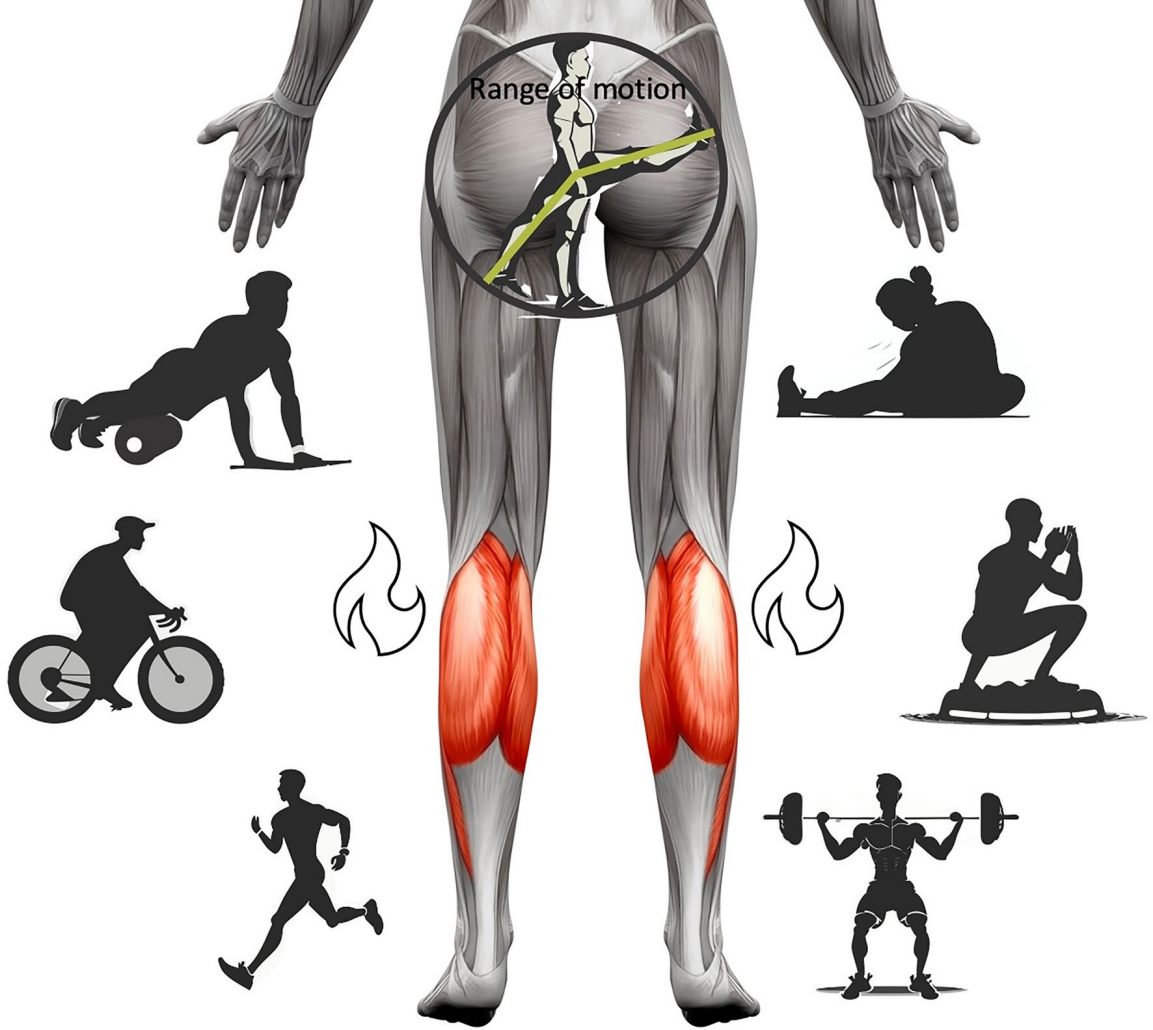
Alternative training routines extracted from the literature that also induced flexibility increases, partially without including stretching. Calf muscles were used as a frequently used example from reviewed studies

To show the necessity of including specific interventions to warm-up routines, a superior effect of stretching or foam rolling compared with alternative routines must be assumed, which cannot be investigated by studies with passive control conditions. Comparing and contrasting stretching and foam rolling responses to the effects of jogging [77], cycling [78], resistance training [60], heat applications [79, 80] or cryotherapy [81], Warneke et al. [82] were not able to confirm the necessity of using specialised routines to acutely enhance flexibility or passive peak torque. While the authors hypothesised a thermal (e.g. thixotropic) effect with general activity-related warm-ups, almost no study assessed muscle and/or core temperature after these routines. Thus, the magnitude of the influence remains speculative. Even though dynamic warm-up effects can contribute to ROM increases, some studies indicated superior effects when using full ROM movements compared with partial ROM [61, 64, 65]. Full ROM movements may provide additional specific factors such as stretching tension, and greater contributions of eccentric contractions. However, the current literature typically did not use appropriate methods to clearly attribute the acute ROM increases solely to foam rolling, stretching, vibration training or other modalities. As only two studies included temperature measurements in response to stretching [76] and foam rolling [83], both confirming increased temperature, observed effects might be attributable to general dynamic warm-up effects. Thus, highly specific explanatory approaches remain speculative and without sufficient evidence, especially when speculating about contralateral and global acute ROM effects of stretching [84, 85] and foam rolling [86, 87] (e.g. influence of myofascial chains) [88]. The available research designs do not allow specific conclusions and should further control approaches to investigate the role of warm-up effects when explaining variance (e.g. temperature), as no further parameters and explanations were controlled to rule out warm-up effects, which could also explain global flexibility enhancements (see Fig. 1).

Thus, we do not doubt the high complexity of physiological mechanisms contributing to acute flexibility increases in response to stretching and foam rolling [17, 47] and are aware that these cannot simply be attributed to general warm-up effects. However, future research designs should consider further approaches.

## Chronic Flexibility Effects

Stretching might be the most common training intervention when aiming to improve flexibility long term. In 2018, Thomas et al. [28] initially asserted the superior effectiveness of static stretching. However, in 2023, Konrad and colleagues [89] updated this perspective by including PNF stretching (in addition to static stretching) as one of the most effective stretching interventions, surpassing dynamic and ballistic stretching. Dynamic stretching demonstrated moderate-magnitude effect size improvements (*d* = 0.55), while static and PNF stretching induced larger magnitude effect sizes (*d* = 1.005 and 1.283, respectively) [89]. As described above, full ROM resistance training might be considered as dynamic loaded stretching [59], which seems interchangeable with stretching routines [5, 59, 90].

Chronic foam rolling effects were explored in a recent meta-analysis that included 11 studies with intervention periods of at least 4 weeks. The results indicated large magnitude increases with an effect size of = 0.823 in joint ROM in the hamstrings and quadriceps, while ineffective for the ankle dorsiflexors [12]. Nevertheless, the results conflict with those of other meta-analyses performed by Pagaduan et al. [91] and Grieve et al. [92] that revealed conflicting and no improvements in response to foam rolling, respectively. Similar to stretching, there is an ongoing discussion regarding the underlying mechanisms of foam rolling. While chronic stretch-induced ROM enhancements were attributed to a combination of structural adaptations (muscle and/or tendon properties) [25, 93, 94] and neuronal changes such as changes in the stretching pain threshold [21–23, 95], foam rolling studies generally agree in attributing ROM increases to enhanced pain tolerance rather than stiffness changes [96, 97]. Non-significant effects were discussed to be attributable to the low-volume interventions of the studies [12, 16].

The literature shows several interventions to induce similar flexibility increases that were accompanied by an overlap in contributing factors such as stiffness decreases, as well as pain threshold increases. Therefore, the question arises about the shared underlying physiology of the listed intervention.

### 3.1. Mechanical Tension with Extended Muscle Lengths as a Possible Underlying Cause for Long-Term ROM Increases—Strength Training or Stretching?

When aiming to improve flexibility long term, it is necessary to perform regular training routines for at least a period of weeks. Undoubtedly, stretch training is one of the most frequently used and investigated training methods to induce ROM improvements [3, 9, 89, 98–102]. In contrast to acute effects, there is little evidence demonstrating chronic ROM increases in response to foam rolling [12], opposed by research pointing out unclear evidence [16, 91, 96]. Possible long-term effects of foam rolling were attributed to the possibility of traction-induced soft-tissue release [16], which is, in fact, not evidence based, while there seems insufficient evidence to support foam rolling as an effective method to release myofascial restrictions [47]. Consequently, Konrad et al. [16] requested further research to address foam rolling-specific underlying mechanisms. Based on contrasting results from available systematic reviews, we decided to focus on training methods with clear evidence instead of speculating about underlying mechanisms of interventions with questionable effectiveness. As Alizadeh and colleagues suggested resistance training to be considered a type of dynamic (loaded) stretching, they pointed out resistance training as a valid alternative to stretch training, showing similar flexibility increases in the long term [5, 59, 90, 103]. Even though it is not clarified if the eccentric portion of full ROM resistance training or the mechanical tension in extended muscle lengths can be considered the underlying mechanisms, stretching alone seems sufficient to induce similar adaptations compared with full ROM resistance training, which is not limited to flexibility changes. While resistance training is recognised for promoting maximal strength gains and hypertrophy, Arntz et al. [104] and Warneke et al. [103] highlighted strength improvements and hypertrophy resulting from stretch training. Notably, stretch training at longer muscle lengths appears to be more effective in inducing muscle hypertrophy [105]. Even though meta-analyses reported smaller magnitude effects of stretching, the results lead us to the question about similarities in the stimulus as well as the underlying physiology.

While not frequently discussed in the stretching literature, a plethora of studies reported the high relevance of mechanical tension in resistance training. Interestingly, when aiming to increase flexibility via resistance training, Alizadeh et al. [59] reported a high relevance of the eccentric portion when performing resistance training [106, 107]. Thus, it can be hypothesised that mechanical stretching would result in changes in the sarcomere length beyond the tissues’ tolerance [108]. More specifically, it has been suggested that these improvements can be related to the remodeling of the extracellular matrix responding to a sarcomere stretch [109]. When comparing ROM effects of 1 h of daily stretching with a commonly performed resistance training (5 × 12 repetitions on 3 days per week for 6 weeks) on muscle thickness, maximal strength and flexibility in the plantar flexors, Warneke et al. [103] showed no significant difference in flexibility increases and hypothesised similarities in the outcomes could possibly be attributed to mechanical tension applied to extended muscle lengths. Assuming that full ROM resistance training and stretching to be sufficient to induce suprathreshold mechanical stimuli in high muscle lengths, the biochemical responses caused by a mechanical overload and its potential impact on ROM enhancements will be discussed in the following.

While it is well established that, in general, skeletal muscle adapts to different mechanical external loads influenced by molecular, subcellular and cellular adaptations [110–113], many specific aspects remain unknown. While numerous animal studies have investigated the stretch-induced protein synthesis enhancements and its impact on cross-sectional hypertrophy of the muscle, the evidence for longitudinal hypertrophy (increased muscle length) is comparatively limited [114]. These studies frequently reported muscle mass enhancements [115–117] and confirmed the stimulation of the myofibrillar protein synthesis rate after inducing mechanical tension via stretching [118–120]. Partially, the enhanced muscle volume was attributed to an increased serial sarcomere number, which was reported in animals after only a few days [121–124] with a consistent large magnitude effect of *d* = 3.31, 95% confidence interval 1.52–5.09, 26.1 ± 7.3% [125].

On a subcellular basis, it can be assumed that stretching causes lengthening of a contractile filament by increasing the distance between the origin and muscle attachment. This position seems to provide a disadvantaged position for contraction due to lower myosin-actin cross-bridge potential while unfolded titin filaments in stretched positions [126, 127] could increase contractile unit tension. Assuming this stretched muscle position to cause a stress stimulus underlines the impact of mechanical overload, which could be achieved via stretching. Herring et al. [128] pointed out that a high “sarcomere number is adjusted so as to achieve an optimum sarcomere length when the muscle is experiencing a high level of tension “, which is stated as the most likely hypothesis. Morphological adaptations can be considered as an optimising process to improve contraction properties. In a given muscle length, increasing the number of sarcomeres in series can be hypothesised to reduce the stretch tension per sarcomere as a suggested underlying mechanism [59, 103, 124], leading to a higher degree in the overlap of the contractile filaments. Accordingly, Zöllner et al. [129] described sarcomerogenesis [114] as a response of skeletal muscle [123] to gradually return into its optimal operating regime after experiencing a stretch beyond the normal physiological limits. This dynamic adjustment can be assumed to be a key factor in long-term regeneration and repairing processes [113, 129, 130]. By inducing a chronic stress situation, Wisdom et al. [110] assumed chronic overstretch [126, 131] and eccentric exercise [108, 132] to be responsible for adding sarcomeres in series, thus increasing muscle length. In contrast, in cats [133], immobilising a muscle in a shortened/flexed joint position caused a decrease in sarcomeres in series and muscle shortening [134, 135]. Both mechanisms were speculated to optimise the pre-condition for contraction by maximising actin-myosin crossbridge potential. Apart from serial sarcomere accumulation [112, 123, 126, 135, 136], longitudinal hypertrophy could also occur in the muscle fascicle [130]. It seems that mechanical tension is sufficient to induce significant morphological changes in skeletal animal muscles [112, 113, 118, 133, 135–137], thus not exclusively important to enhance the muscle cross-sectional area, but also the muscle length (serial sarcomere number).

Moreover, as described above, increasing the distance between Z-discs and the subsequent titin unfolding, which can be assumed by adopting large degrees of stretching, could be of paramount importance for structural muscle adaptations. Van der Pijl et al. [138, 139], Linke [140], and Freundt and Linke [141] discussed the relevance of titin unfolding for stimulating signalling pathways with changes in the protein synthesis rate as an important contributor of the sarcomerogenesis [142]. Thus, it seems reasonable to discuss the role of mechanical tension for triggering anabolic signalling pathways as a part of morphological adaptations via stretching. Accordingly, assuming the unfolding of titin is exclusively attained by reaching long muscle lengths (consequently maximise mechanical tension via stretch), Apostolopoulos et al. [143] pointed out the high relevance of stretching intensity when aiming to induce structural changes. The stimulation of the signalling pathways leading to protein synthesis is discussed following the response matrix model by Toigo and Boutellier [144].

### Response Matrix Model

Suprathreshold external stimuli (such as mechanical tension) were described to lead to a specific gene expression, which takes place by transcription and translation of specific proteins [145] causing a specific biological response that results in specific changes in the phenotype. So far, based on aforementioned results from animal experiments, a serial sarcomere accumulation due to a high mechanical stress applied to a high muscle length seems reasonable. When adding sarcomeres in series, similar to cross-sectional hypertrophy, new muscle protein must be synthesised via anabolic processes that stimulate specific protein synthesis, which can be triggered via a stretch-induced mechanical overload [120, 146–148]. Thus, it is hypothesised that longitudinal hypertrophy might be also initiated via mechanotransduction, which describes the translation of mechanical signals to biochemical responses [149, 150].

**Fig. 2:**
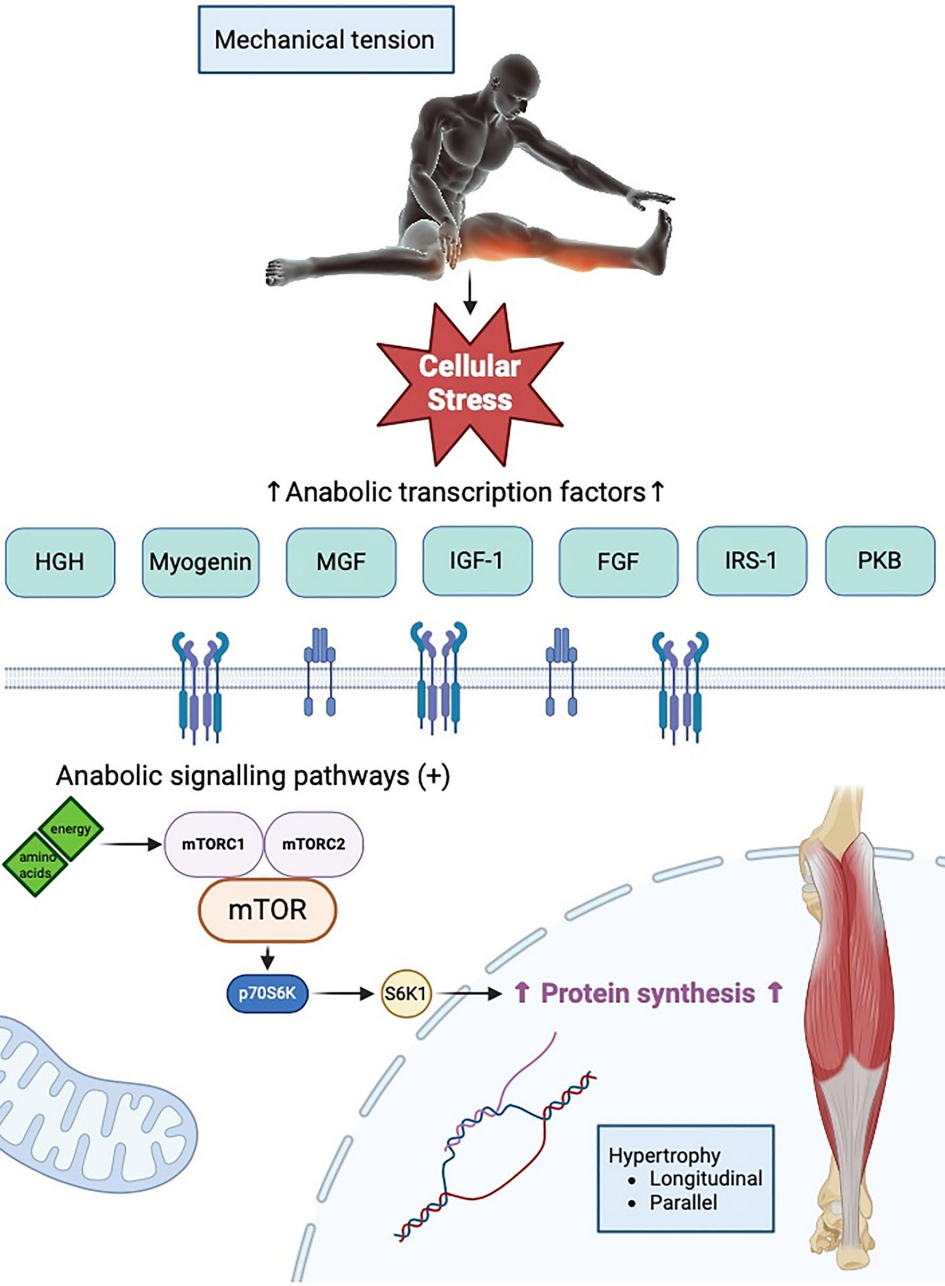
Simplified illustration of the most popular signalling pathway to induce anabolic adaptations (illustrated with Biorender.com). *FGF* fibroblast growth factor, *HGH* human growth hormone, *IGF-1* insulin-like growth factor-1, *IRS-1* insulin receptor substrate 1, *MGF* myogenin growth factor, *PKB* protein kinase B. Created with BioRender.com

In line, referring to Coffey and Hawley [151], converting a mechanical signal generated during contraction to a molecular event promotes adaptations in a muscle cell that might cause a subsequent upregulation of primary and secondary messengers. These mechanotransduction mechanisms [152] compromise the translation of mechanical stimuli such as stretching via, among others, stretch-activated channels to biochemical responses via signalling pathways, which in turn adapt the muscle in length to reduce the stretching stressor [149, 153]. The addition of serial sarcomeres, thus producing additional proteins is the response to a specific muscle protein synthesis enhancement. To initiate this anabolic response, several known signalling cascades are stimulated that result in an activation and/or repression of specific signalling pathways to regulate gene expression and protein synthesis/degradation in response to training [120, 152]. Even though the exact downstream events of the anabolic signalling need further investigation [154], there seems to be a consensus that mechanical tension induced via resistance training or stretching [148] triggers specific signalling pathways. More specifically, the Akt/mTOR/p70S6K is described to play a fundamental role in muscular anabolism [113, 155–157]. Interestingly, several transcription factors such as insulin-like growth factor-1 [113, 158–160], myogenin growth factor, insulin receptor substrate 1 and protein kinase B [161] playing an important role by activating the anabolic mTOR pathway [150, 162] are activated by stretching [113, 120, 133, 136, 150, 163] as well as resistance training [155, 156, 164] (Fig. 1). Wang and Proud [165] described further anabolic events, including PIP3, PKB and Tuberous sclerosis 1 and 2 [119, 160, 165]. Assuming an increased net protein synthesis rate as important, it is notable that tension applied via stretching can cause a downregulation of anti-anabolic or catabolic mediators such as myostatin, MuRF1 or MAFbx via the insulin-like growth factor-1-induced inhibition of FOXO [112, 113, 166]. Assuming specific adaptations, it seems reasonable that sarcomerogenesis also occurs in human muscles as a response to mechanical tension induced in long muscle lengths—independent of the way this mechanical stressor is induced (full ROM resistance training, dynamic stretching, static stretching, PNF stretching). Figure 2 shows a simplified illustration of the most popular signalling pathway to induce anabolic adaptations.

Even though the human literature favors neuromuscular stretching adaptations, as structural adaptations were described unlikely [21, 22], it seems questionable to separate neuromuscular from structural adaptations. Considering stretching as a physiological response, warning the body of possible harm, injury or chronic pain to trigger appropriate protective responses [167, 168], increases in the functional muscle length might cause a better joint stabilisation in the end ROM, thus causing a later myotatic reflex occurrence with increases in the stretching pain threshold.

### Methodological Problems Limiting the Evidence

Yet, although logical in theory, no direct evidence could be found for serial sarcomere accumulation in humans. On the one hand, this fact might be attributable to methodological concerns in data collection. On the other hand, there is a lack of studies inducing a meaningful stretch stimulus that was sufficient to induce serial sarcomere accumulation in animals.

It is speculated that serial sarcomere accumulation occurs in humans; however, it is complicated to directly determine this phenomenon. In animal studies, the effectiveness of the stretching protocol is examined by extracting a muscle sample following the euthanasia of the subject. This direct analysis, however, poses limitations as researchers are confined to post-intervention samples, making within-subject comparisons impossible. Consequently, many direct analyses resort to employing two separate groups, introducing potential challenges for generalisation [125, 169]. It seems impossible to discuss the transferability of such a study protocol to human participants. In humans, the limited data are therefore attributable to a limited possibility to directly measure the serial sarcomere number directly. While invasive methods (i.e. biopsies) might be applicable in humans, it is not possible to perform a direct pre-post testing, as the same muscle tissue cannot be removed twice. Even for those methods, data collection and probe evaluation equipment such as biopsies or microendoscopy [170, 171] would be necessary, which seems of limited accessibility.

However, indirect evidence for stretch-induced structural adaptations as well as the underlying physiological explanations is limited. While most of the discussed mechanisms were discussed in the resistance training literature, there is evidence for many similarities in stretching [5, 118, 146, 160, 163, 172]. Assuming high mechanical tension to induce structural damage with subsequent increases in protein synthesis, creatine kinase activity is commonly determined as a valid predictor [173–175]. Even though Smith et al. [176] indicated that static stretching (3 × 60 s of stretching with 17 exercises) was sufficient to increase creatine kinase activity compared with ballistic stretching, the clinical relevance of such low magnitude creatine kinase improvements was questioned [177]. Indeed, Fowles et al. [178] did not find a significant protein synthesis increase in response to 33 min of plantar flexor stretching. Nevertheless, stretching durations (and volumes) in animals with 24 h per day, which induced a meaningful protein synthesis increase, were not comparable to those used in humans (up to 33 min) [178], especially when considering a slower protein synthesis rate in humans [179, 180], compared with chickens and quails as well as to rats and cats [181, 182]. Indeed, small stretching volumes and durations were not sufficient to increase the muscle cross-sectional area [183], while higher volumes (up to 7 h per week) consistently showed muscle hypertrophy [146].

Nevertheless, although serial sarcomere accumulation was not measured directly in humans, there are some indications such as increased ROM [3, 9, 98–102], reduced pain threshold [21–23, 95], changes in the optimal force production angle or strength increases in long muscle lengths [184–186] as well as changes in fascicle lengths [187] after performing long-term stretching programmes. These adaptations, however, were not exclusively related to stretching exercises, but also to resistance training. In accordance with Alizadeh et al. [59] emphasising the role of the eccentric phase of resistance training, Gérard et al. [188] reviewed the available eccentric resistance training literature and indicated significant pennation angle decreases, while Marušič et al. [189] showed 6 weeks of an eccentric hamstrings training protocol, confirming pennation angle decreases that were accompanied by fascicle length increases, which could indirectly underline muscle length changes.

However, based on similarities in the stated physiological responses as well as outcomes, it seems reasonable that a mechanical overload applied to long muscle lengths would optimise training-induced ROM adaptations also in humans. In line, similar results using high-volume stretching and strength training [5, 59, 90, 103, 104] could possibly be explained by these shared underlying mechanisms. Although stretching intensity is stated to be of paramount importance to induce structural adaptations [143], intensity was mostly measured using subjective pain perception. To therefore optimise training studies and methods in sports practice, conducting well-designed stretching studies including objective intensity (tension) quantification as well as physiological outcomes seem imperative, while there is a limited need for further studies confirming the influence of stretching on flexibility.

### Further Potential Mechanisms

Even though frequently reported to be of high importance, a mechanical overload seems not the only stimulus to induce structural muscle adaptations. Considerable structural adaptations were reported in response to blood flow restriction [190], which were potentially initiated by similar physiological pathways [191] including the mTOR signalling pathway [192], with Gundermann et al. [193] showing stimulation of mTORC1 and muscle protein synthesis immediately after blood flow restriction training. Interestingly, in the rat muscle, Hotta et al. [194] described an almost complete suppression of the blood flow to the stretched muscle. Thus, potential stimulating effects of restricted blood flow on structural adaptations cannot be ruled out, while stretch-induced changes in blood flow patterns in human muscle have not been comprehensively investigated, while no studies were found that showed an increased flexibility in response to blood flow restriction training. Furthermore, metabolic factors (accumulation of metabolites due to suppressed blood flow) might induce stress that could increase structural adaptations in general. The link to increased flexibility, however, remains uninvestigated and will therefore not be further discussed here.

## Practical Applications in Sports Practice and Research

Referring to Alizadeh et al. [59], it is not mandatory to perform stretching, as resistance training performed over the full ROM resulted in similar adaptations. Assuming muscle tissue tends to optimise the myosin and actin overlap [128], structural adaptations can be hypothesised to be influenced by adding a contraction to the stretch. Accordingly, in animal research, Williams et al. [133] showed in cats that immobilisation of the hindlimb muscle in a stretched position increased the number of sarcomeres in series significantly, while immobilisation in a shortened position showed a significant decrease in the number of sarcomeres in series. In humans, adding electrostimulation to a stretched position causing a contraction of the muscle in high muscle lengths might be an important further stimulus [195]. Interestingly, most recently, the practical relevance was confirmed by Mizuno [40] showing superior effects regarding ROM, passive torque adaptations and tendon displacement by adding electrostimulation to static stretching in the calf muscles.

Thus, assuming greater degrees of mechanical tension with long muscle lengths to be one important underlying mechanism to induce structural muscle adaptations, applying an active contraction in stretched muscle positions can be hypothesised to enhance the stress stimulus (muscle contraction with a minimal actin-myosin overlap). Therefore, when aiming to increase active ROM, there might be different ways to induce high degrees of mechanical tension to greater muscle lengths. Considering full ROM resistance training as a type of loaded dynamic stretching would be one way to include active movements over the available muscle lengths. However, assuming the time under tension to be important as well [196], the exclusive focus on high-intensity (thus low volume or duration) resistance training could be reasonably supplemented by high-volume stretch training. Nevertheless, stretching is still of high relevance to enhance ROM long term. Full ROM resistance training such as deep squats are multi-joint exercises that might compensate, for example, limited angle joint flexibility by flexing the hip joint and bending the upper body, thus avoiding the weakness without improving ankle ROM. Furthermore, improving adductor flexibility might be difficult when using complex multi-joint movements. Therefore, based on the outlined literature, it can be recommended to use isolated stretching exercises to improve individual joint ROM, while full ROM resistance training could be considered a more economical method to induce high degrees of mechanical tension in longer muscle lengths, thus improving full-body active flexibility long term comparable to stretching [59, 90].

In contrast, when aiming to acutely enhance flexibility, there is no evidence to emphasise specific interventions, as no significant differences in interventions can be assumed. As warm-up routines should avoid fatigue and should be limited to essential movement preparation, in contrast to the common literature, the inclusion of stretching or foam rolling might be considered a waste of time, as almost every concurrent intervention (jogging, cycling) would induce similar ROM increases, however, by performing active and more sports-related movements, in a best case by using full ROM [61, 64, 65].

When conducting research designs, but also for training interventions in sports practice, it seems more appropriate to focus on physiological aspects. Therefore, training can be seen as an external stressor, resulting in specific physiological adaptations to cause structural and functional changes of the system. To reasonably perform training interventions, in the first step, it seems valuable to be clear about the underlying system and the type of the stimulus to cause a suprathreshold stimulus (mechanical stress on the muscle cell in high muscle lengths). Afterwards, considering the available training methods, the practitioner has to choose the methods, inducing the highest amount of stress to the targeted system.

## Conclusions

In this review, we discussed explanatory approaches for acute and chronic flexibility increases, which contrast with common theories. Several limitations in the research design of the currently available research do not allow the conclusion of specific acute stretching results, as several interventions showed comparable ROM effects. Additionally, for long-term effects, ROM can be enhanced by several other interventions than stretching, which induce mechanical tension to longer muscle lengths, which does not mean that this stimulus is the only mechanism. Nevertheless, the favored approach is the role of mechanical tension, which might induce several adaptations, such as stretch-induced sarcomerogenesis, as an increased muscle length would also delay the occurrence of stretching pain and reduced stiffness as frequently reported accompanying effects.
